# Editorial: Identification and characterization of molecular targets in hepatocellular carcinoma

**DOI:** 10.3389/fmolb.2023.1202614

**Published:** 2023-06-26

**Authors:** Vinay Kumar, Surendra Kumar Shukla

**Affiliations:** ^1^ Department of Physiology and Cell Biology, Dorothy M. Davis Heart and Lung Research Institute, The Ohio State University Wexner Medical Center, Columbus, OH, United States; ^2^ Department of Oncology Science, OU Health Stephenson Cancer Centre, Oklahoma City, OK, United States

**Keywords:** hepatocellular carcinoma (HCC), immunotherapy, metabolic alterations, gene network, HCC prognosis, liver cancer

Hepatic malignancies remain a global challenge with an increasing disease burden worldwide. According to an estimate by 2025, more than 1 million people will be affected by it annually (Llovet et al., 2016), GLOBOCAN 2018. *IARC).* Hepatocellular carcinoma is the most common type of liver cancer representing about 90% of all the cases of hepatic malignancy (Llovet et al., 2021), and according to World Health Organization 2020 record, it is the third leading cause of cancer-related deaths worldwide World Health Organization 2020. Most of the HCC-related mortality occurs in Asian and African countries but the incidences of HCC are on the rise in European countries and the United States. According to the report of the SEER (Surveillance Epidemiology End Results), mortalities related to HCC are on the rise in the United States (McGlynn et al., 2015), and it will become the third leading cause of cancer-related deaths by 2030 (Rahib et al., 2014). Along with viral infection (Hepatitis B Virus-HBV and Hepatitis C Virus-HCV) metabolic diseases such as non-alcoholic steatohepatitis (NASH), diabetes, and obesity are considered key risk factors for HCC development (Global Burden of Disease Liver Cancer et al., 2017; Gupta et al., 2018). The molecular mechanism of HCC pathogenesis varies depending on the genotoxic insults and altered physiological conditions of the body. The advancement of different molecular and biochemical technologies to explore the disease pathogenesis at the cellular level as well as in a systemic way provides plenty of opportunities to explore novel biomarkers of disease progression and identify new therapeutic targets.

Metabolic alterations are considered a hallmark of cancer (Hanahan, 2022). Tian et al., by utilizing *in silico* and *in vitro* studies, have shown that patients with higher expression of key metabolic enzymes such as *LDHA* and *CHAC2* exhibit poor survival. However, higher expression of some metabolic enzymes like *ADPGK*, *GOT2*, *MTHFS*, and *FDCD* was associated with a better prognosis. They have also established the correlation between metabolic alteration and the TP53 mutation rate. In a similar line of studies, Zhou et al., have demonstrated that expression of tyrosine metabolism-related genes (TRGs) mainly*: METTL6, GSTZ1, ADH4, ADH1A, and LCMT1* can be utilized as a predictor of HCC patient’s prognosis. Yang et al., have demonstrated the role of HIF2-alpha, a key metabolic regulator, along with VEGF in cellular proliferation and migration of HCC cells in response to insufficient radiofrequency ablation. Angiogenesis plays a very important role in the pathogenesis of several types of cancer including HCC. Tang et al., have shown that expression of angiogenesis-related genes (ARGs) exhibits predictive value in the prognosis of HCC patients. They identified about differential expression of 97 ARGs and further constructed 9 genes-based models to predict the prognosis of HCC. Tang et al., have demonstrated the importance of senescence-related genes in the prognosis of HCC patients. Their study demonstrates that cellular senescence-related genes can be utilized as a prognostic marker as well as a biomarker of therapeutic response. These studies altogether establish the importance of metabolic alterations related to gene expression and angiogenesis in the prognosis of HCC patients.

Epigenetic modifications regulate several aspects of cellular physiology and different disease progression by regulating the gene expression machinery. The role of epigenetic alterations in the pathogenesis of different types of cancers including HCC is well-established but not completely understood. By utilizing the publicly available databases, Wang et al., have identified that genes related to N^6^-methyladenosine (m6A): B2M and SMOX can serve as prognostic signature and their expression may guide to design of novel therapeutic strategies for HCC patients. In a similar study, Huang et al., have demonstrated a 12 genes-based risk signature model in the prognosis of HCC patients. They have shown that SDC3, NCF2, BTN3A3, and WARS genes can serve as novel prognostic factors for HCC.

Immune therapeutics are revolutionizing cancer treatment, so it’s high time to identify the novel immune modulators of HCC pathogenesis and potential immune therapy targets. Wang et al., have established a correlation between mitophagy-related genes and immune infiltration in a subset of HCC patients. They have shown that a subset of patients exhibiting higher mitophagy-related gene expression show poor prognosis and suppressed immune function. In another study, Qu et al., have shown that M2-like macrophage markers like PAM and LGALS3 expression positively correlate with the sensitivity of simvastatin and ARRY-162. Further, they have predicted ten anticancer drugs with higher sensitivity towards the high-mitophagy gene expression group. Further, Cao et al., have shown that March ligases expression regulates immune cell infiltration in HCC tumors. By utilizing the TCGA data set of liver cancer patients, Wang et al., have shown that genes related to copper metabolism correlate with immune infiltration in HCC. Their finding may be very useful in establishing the immunotherapy response biomarker. Liang et al., have shown that ferroptosis regulator membrane protein SLC7A11 exhibits the highest expression correlation with the immune checkpoint gene PD-L1. They have also established that SLC7A11 can serve as an independent prognostic signature itself for HCC patients. Furthermore, another study by Zhang et al., has shown that ferroptosis-related genes were significantly correlated with tumor immune infiltration and immune checkpoint genes expression. Long et al., have also explored the correlation of immune regulatory genes with HCC patients’ survival. Their study has demonstrated that 5 immune regulatory genes expression has significant predictive importance in HCC patients’ survival. In a review article Si et al., have discussed the importance of IL32 and IL34 expression in HCC pathogenesis and therapeutic targeting.

In general authors of these articles have done molecular characterization of HCC patient samples gene expression, and immune infiltration and studied their impact on the prognosis of HCC patients. Some studies have explored the correlation between gene signatures and therapeutic response along with their prognostic values. The role of metabolic alteration-related genes, angiogenesis-related genes, and genes involved in different types of cell death such as cuproptosis and ferroptosis have been shown to possess prognostic value and correlate well with the different immune phenotypes of HCC tumors. A key limitation of most of the studies is that results are based on purely *in silico* analysis of publicly available databases, and very limited validation has been done in laboratory conditions. Most of the studies are correlative, so it will be premature to conclude their direct role of a predicted gene signature in HCC pathogenesis. More preclinical and clinical studies are needed to validate these findings.
